# Single incision thoracoscopic right upper lobectomy with systematic lymph node dissection

**DOI:** 10.1186/1749-8090-9-66

**Published:** 2014-04-04

**Authors:** Hyun Woo Jeon, Young-Du Kim, Young Kyu Moon, Young Pil Wang

**Affiliations:** 1 Department of Thoracic and Cardiovascular Surgery, Bucheon St. Mary’s Hospital, Gyeongigido Sosaro, Wonmi gu, Bucheon city 420-717, College of Medicine, The Catholic University of Korea, 222 Banpo-daero, Seocho-gu, Seoul 137-701, Republic of Korea; 2 Seoul St. Mary’s Hospital, College of Medicine, The Catholic University of Korea, 222 Banpo-daero, Seocho-gu, Seoul 137-701, Republic of Korea

**Keywords:** Video-assisted thoracic surgery, Adenocarcinoma of lung, Mediastinal lymph node dissection

## Abstract

Video-assisted thoracic surgery (VATS) provides less postoperative pain, preservation of the immune response and shorter recovery period, compared with thoracotomy. However, many patients complain of postoperative pain and paresthesia because VATS requires 3 or 4 incisions including a utility incision of 3–5 cm. To overcome this problem, single incision thoracoscopic surgery has emerged; this technique has been adopted for lung cancer surgery since 2010. Complete mediastinal lymph node dissection is the major role of lung cancer surgery. We describe a case of a right upper lobectomy with complete mediastinal lymph node dissection via single incision thoracosopic surgery.

## Background

Since the 1990s, VATS lobectomy has been a popular procedure for non small cell lung cancer (NSCLC). It provides reducing postoperative pain and early recovery; in addition, oncologic results are comparable to those of a thoracotomy [1]. Because VATS usually creates 2 or 3 ports with a utility incision (3–5 cm). Paresthesia and pain commonly occur [2]. Since 2010, Single incision VATS lobectomy has been performed for lung cancer. Lobectomy for lung cancer includes two major procedures (anatomical division of the lobar artery, vein and bronchus, as well as complete mediastinal node dissection). Major concern is whether complete lymph node dissection is possible through only small incision. The literature lacks reports of right sided mediasitnal lymph node dissection using single incision VATS.

## Case presentation

**Figure 1:**
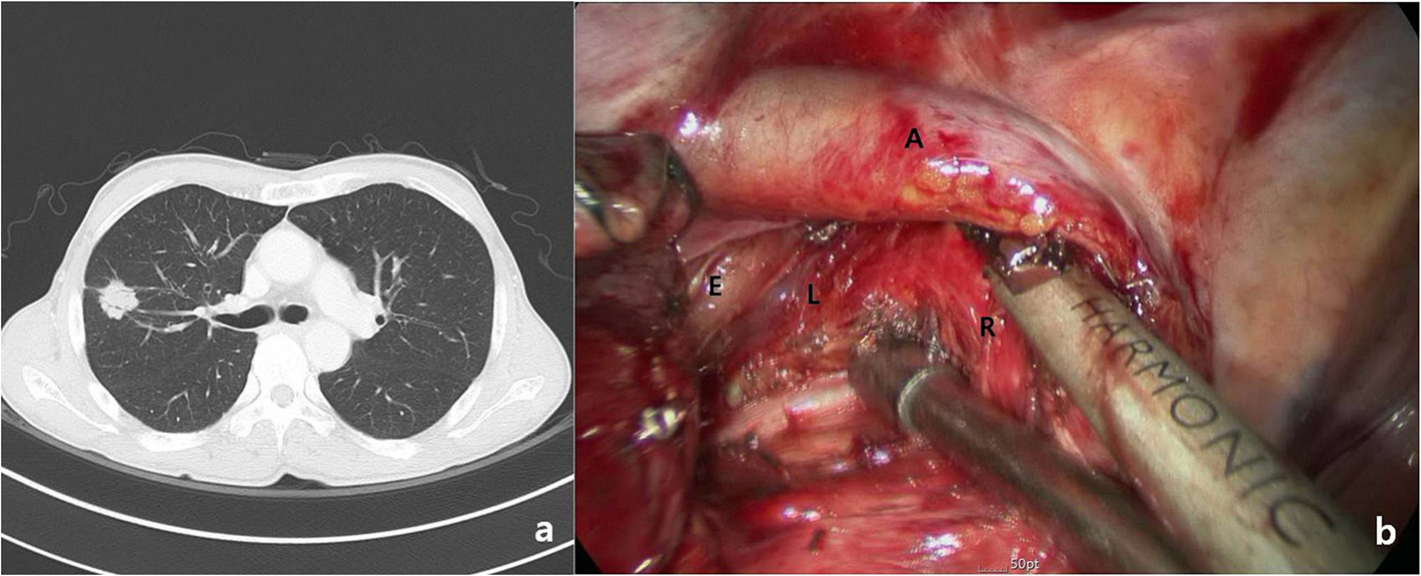
**Chest computed tomography revealed 1.8 cm nodule with spiculation in Right upper lobe (a) Subcarinal lymph node dissection (b) bounded by right main bronchus (R) and left main bronchus (L).** A: Ayzgos vein, E: Esophagus.

A 70 year old Korean man was referred for abnormal chest radiograph. He underwent a coronary stent for angina nine years ago and suffered from diabetes mellitus and hypertension. A Chest computed tomography (CT) showed a 1.8 cm nodule with spiculation in the right upper lobe (RUL) (Figure 1a). Preoperative assessment included brain magnetic resonance imaging (MRI), bone scanning, positron emission tomography (PET)-CT, Echocardiogram and bronchoscopy. Malignancy was suspicious and the clinical stage was T1aN0M0; therefore, we planned thoracoscopic surgery without obtaining a tissue diagnosis.

### Surgical technique

**Figure 2:**
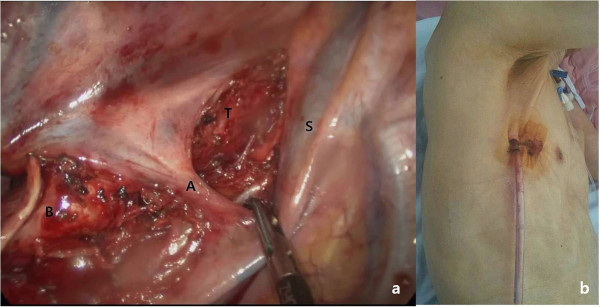
**Single port thoracoscopic mediastinal node dissection. (a)** Dissection for Upper mediastinal nodal stations bounded by Trachea (T), superior vena cava (S), stapled upper lobe bronchus (B) and azygos vein (A). 4cm skin incision was made in the 5^th^ intercostal space at the anterior axillary line for right upper lobe lobectomy with complete lymph node dissection **(b)**.

Under general anesthesia, the patient was placed in left lateral decubitus position. A single 4 cm incision was made in the 5^th^ intercostal space at the anterior axillary line. Wound protector (Applied Medical, Rancho Santa Margarita, CA, USA) was applied for prevention of contamination from lung cancer. After selective one lung ventilation, incomplete fissure was found under 30 degree-10 mm scope. The RUL mass was identified by digital palpation. The first step of the procedure was dissection of the interlobar space with a Harmonic scalpel (Ethicon Endo-Surgery, Inc., Cincinnati, Ohio, USA); then we identified the posterior segmental artery of the RUL and superior segmental artery of the right lower lobe (RLL). The posterior portion of the major fissure was dissected and tunneled followed by division using a endostapler (TriStapler, Covidien, Norwalk, CT). The RUL was retracted anteriorly and a scope was placed at the posterior portion of incision. The RUL bronchus was identified and peribronchial tissue was cleared. The RUL bronchus was encircled with a long right angle clamp with a silastic drain followed by division using a endostapler. The RUL was retracted to the caudal portion. Truncus anterior branches were easily dissected and divided. The RUL was retracted to the costophrenic angle. The superior pulmonary vein was identified and tunnel was created using a right angle clamp with a silastic drain. The vein was divided. To the caudal retraction of RUL, posterior segmental artery was easily exposed and divided. Due to the incomplete minor fissure, the fissure was created using endostaplers (Additional file 1: Video 1). Inferior pulmonary ligament division was carried out. During this procedure, the scope was placed at the anterior portion of incision and lower mediastinal nodal stations were removed. For subcarinal lymph node dissection, Divided RUL bronchus was retracted to the anterior portion using the endoinstrument or the tip of suction. The scope was placed at the center of the incision. The lymph nodes were grasped using an endoinstrument through the posterior portion of incision. The lymph nodes were removed using Harmonic scalpel through the anterior portion of incision (Figure 1b). Hilar lymph nodes were dissected below the azygos vein. For upper mediastinal node dissection, Azygos vein was elevated. Mediastinal fat tissue including lymph nodes was separated from the trachea and superior vena cava then mediastinal pleura was incised above the azygos vein. En bloc resection was performed including fatty tissue and lymph nodes bounded by the RUL bronchus, subclavian artery, superior vena cava and trachea (Figure 2a) (Additional file 2: Video 2). A chest tube was placed via the posterior portion of the incision and the skin incision was closed with a subcuticular running suture method (Figure 2b). Total surgery time was 150 min. Blood loss was ≤ 50 ml. The chest tube removal was removed when the daily drainage was ≤ 100 ml. The patient was discharged on postoperative day 8 without complications. Intravenous patient controlled analgesia using fentanyl was performed for relief of postoperative pain. Visual analog scale was estimated. Maximum score was 7 (immediate postoperative state). However, Score was 2 on postoperative day 1 and 0 from postoperative day 4. The pathologic diagnosis was adenocarcinoma (2.4×1.4 cm), stage T1bN0M0. The total number of lymph nodes dissected was 38; 30 lymph nodes were obtained from mediasitnal nodal stations.

## Discussion

Although VATS lobectomy has been one of the attractive treatment modalities for lung cancer, the current trend is to reduce pain and minimize paresthesia. Single incision thoracic surgery has attracted the attention of thoracic surgeons. The potential advantages of single incision surgery are reduction in postoperative pain and paresthesia [2,3]. Single incision VATS lobectomy for lung cancer has been performed since 2010 [4]. However, Single incision VATS lobectomy for lung cancer is controversial although it is a safe and feasible procedure for lung cancer [5,6], VATS lobectomy for lung cancer encompasses two major procedures. One is the anatomical division of the affected lobe including lobar artery, vein and bronchus, the other is complete mediastinal node dissection. The literature contains a few studies for single incision lobectomy for lung cancer. Most of them focused on anatomical resection compared with complete mediastinal node dissection.

Since 2011, we have been used the single incision technique for pneumothorax and diagnostic wedge resection. In 2013, we adopted this technique for lung cancer. Initially, we performed this technique in stage IA with complete fissure. In RUL lobectomy with conventional VATS, we prefer the mid-axillary approach. Single incision technique was similar to conventional VATS for RUL lobectomy and the instruments were same. Although a single incision lobectomy has some limitations, (straight instruments with 30 degree scope are placed through a single incision and endoscopic view is compromised), Anatomical lobectomy is feasible. However, complete mediastinal node dissection is difficult. Technical problem is obstruction of view and mutual impingement of instruments. Sometimes, more than four instruments are inserted through the single incision; thus, coordination is difficult so suitable devises for single incision surgery may be necessary. Rivas et al. opened the pleura below the azygos vein and paratracheal lymph nodes were removed during lifting of azygos vein [5]. In conventional VATS, we always incise the square shape of mediastinal pleura bounded by the RUL bronchus, subclavian artery, superior vena cava and trachea. Mediastinal fatty tissuess including lymph nodes are removed according to the European Society of Thoracic Surgeons (ESTS) guidelines [7]. This procedure allows for detection of hidden lymph nodes; thus, it facilitates complete lymph node dissection. Wang et al. reported the safety and feasibility of single incision thoracoscopic lobectomy. Complete mediastinal node dissection (median number of lymph node dissected: 22.9) was achieved without major complications [8].

## Conclusions

Single incision VATS lobectomy for lung cancer could be conducted safely in selected patients and the complete fissure may be a good indication. However, further study will be necessary to apply this technique including safety, oncologic results and advantages or disadvantages.

## Consent

Written informed consent was obtained from the patient for publication of this case report and any accompanying images. A copy of the written consent is available for review by Editor-in-Chief of this journal.

## Abbreviations

VATS: Video-assisted thoracic surgery; NSCLC: For non small cell lung cancer; CT: Computed tomography; RUL: Right upper lobe; MRI: Brain magnetic resonance imaging; PET-CT: Positron emission tomography; RLL: Right lower lobe.

## Competing interests

The authors declare that they have no competing interests.

## Authors’ contributions

HW Jeon carried out review of medical record and writing. YD Kim is corresponding author, carried out revision of the manuscript. YK Moon carried out the review of the medical record. YP Wang carried out revision of the manuscript. All authors read and approved the final manuscript.

## Supplementary Material

Additional file 1 **Video 1.** Lobectomy through the single incision VATS. Click here for file

Additional file 2 **Video 2.** Mediastinal lymph node dissection. After lobectomy, lower mediastinal, subcarinal, hilar and upper mediastinal nodes were dissected. Click here for file
